# Aortic bypass and orthotopic right renal autotransplantation for midaortic syndrome: a case report

**DOI:** 10.1186/1471-2482-14-86

**Published:** 2014-11-05

**Authors:** Hao Zhang, Fang-da Li, Hua-liang Ren, Yue-hong Zheng

**Affiliations:** Department of Vascular Surgery, Peking Union Medical College Hospital, Peking Union Medical College and Chinese Academy of Medical Sciences, No, 1 Shuaifuyuan, Dongcheng district, Beijing, 100730 P.R China

**Keywords:** Midarotic syndrome, Aortoaortic bypass, Renal artery stenting, Orthotopic renal autotransplantation, Hypertension

## Abstract

**Background:**

Midaortic syndrome (MAS) is a rare vascular anomaly characterized by segmental narrowing of the distal descending thoracic or abdominal aorta. Renal or visceral arteries may also be affected to varying degrees. MAS is often associated with renovascular hypertension, and requires early intervention. When medical therapy and percutaneous interventions fail to control hypertension, surgical treatment is required. We report a case of MAS that failed to respond to bilateral renal artery stenting, but treated with aortic bypass and orthotopic right renal autotransplantation with good outcome.

**Case presentation:**

A 31-year-old woman presented with headache and poorly controlled hypertension due to severe MAS. She had severe ostial stenoses of renal and visceral arteries. Her hypertension failed to respond to medical therapy (four drugs) and bilateral renal artery stenting. The implanted stent in the right renal artery rendered revascularization of the artery difficult. A one-stage revascularization was performed, which consisted of an aortoaortic bypass (between the suprarenal and infrarenal abdominal aorta) with a prosthetic graft, an orthotopic right renal autotransplantation and an aorto-left renal arterial bypass with autogenous saphenous vein grafts. Her recovery was uneventful. At 1-year follow-up, the patient remained well. Her hypertension improved. A postoperative computed tomography angiography showed that all the grafts were patent with no abnormalities at the anastomosis.

**Conclusion:**

Multiple bypass surgery with reimplantation of autogenous vein graft onto the prosthetic graft is a feasible and effective procedure in renal artery revascularization for MAS. Orthotopic autotransplantation is the procedure of choice in complex renal artery reconstruction.

## Background

MAS is a rare vascular anomaly characterized by segmental narrowing of the distal descending thoracic or abdominal aorta. The term midaortic syndrome (MAS) was originally used by Sen et al [1]. MAS may be congenital or caused by some acquired reasons, such as Takayasu's arteritis, neurofibromatosis and fibromuscular dysplasia [2]. Visceral and renal artery stenoses are usually attached to the syndrome. Proximal renal artery stenosis may occur in up to 80% of cases, and visceral arteries may be stenosed in at least 30% of cases [2, 3]. MAS is often associated with refractory hypertension. If left untreated, most patients would die by the age of 40 as a result of complications from hypertension [3]. Computed tomography angiography offers convenient, fast and sharp images, and it is a useful tool in diagnosing MAS. Treatments for MAS include medical management, endovascular therapy, bypass grafting, and patch aortoplasty [2, 4]. Kim et al introduced a novel treatment for midaortic syndrome, in which a tissue expander was used to induce longitudinal growth of the normal distal abdominal aorta and iliac arteries in a 3-year-old girl [5]. The goals of treatment for MAS are to normalize the blood pressure, avoid any complications of the hypertension, and preserve the renal function as much as possible. Invasive measures are favored when medical management is insufficient or there is impending end-organ damage [3].

The present report involves a case of severe MAS affecting the renal and visceral arteries. She had undergone stenting for bilateral renal artery stenoses at another hospital. The patient successfully underwent a one-stage revascularization with an orthotopic right renal autotransplantation in our hospital. Since very few technical reports on orthotopic renal autotransplantation in MAS revascularization are found in the literature, the present report would add significant value.

## Case presentation

**Figure 1:**
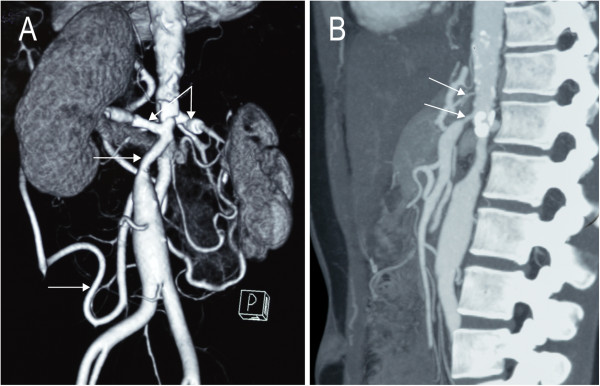
**Preoperative imaging showing multiple arterial stenoses. A**, Posteroanterior view of three-dimensional computed tomography angiography showing coarctation of the abdominal aorta (middle arrow), bilateral renal artery stents (two upper arrows) and arc of Riolan (lower arrow). **B**, Superior mesenteric artery stenosis (lower arrow) and celiac artery stenosis (upper arrow).

A 31-year-old Chinese woman presented with headache and poorly controlled hypertension. Her blood pressure was 170/100 mm Hg in the brachial artery and 150/90 mm Hg in the thigh. She had a history of hypertension for about 20 years and her medical therapy comprised four antihypertensive medications (hydrochlorothiazide, amlodipine, arotinolol, irbesartan). Two years previously, she was diagnosed with Takayasu's arteritis and had undergone stenting for bilateral renal artery stenoses at another hospital. However, her arterial hypertension failed to improve. In the present admission, laboratory investigations revealed that a blood urea nitrogen level of 26.2 mg/dl, serum creatinine level of 4.9 mg/dl and erythrocyte sedimentation rate of 7 mm/h. Technetium-99m-diethylenetriaminepentacetic acid (99mTc-DTPA) renogram revealed decreases in the renal blood flow and the glomerular filtration rate (GFR). The GFR of the left and right kidney were 20.4 ml/min and 12.1 ml/min, respectively (total GFR 32.5 ml/min). Computed tomography angiography showed marked narrowing of the abdominal aorta, stents in bilateral renal arteries, well-established arc of Riolan, and ostial stenoses of the superior mesenteric artery and the celiac artery. Her right kidney was significantly atrophied (Figure 1). The thoracic aorta and its branches were normal.

**Figure 2:**
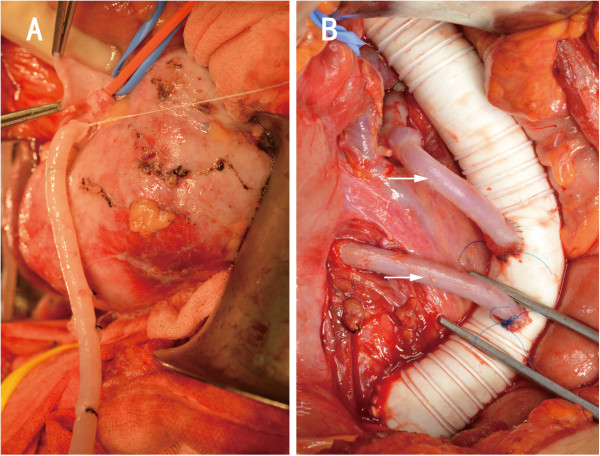
**Cold perfusion of the right kidney and multiple bypass surgery. A**, Cold perfusion of the right kidney with University of Wisconsin solution. **B**, Autogenous saphenous vein bypasses connect the left (upper arrow) and right (lower arrow) renal arteries to the prosthetic graft.

Finally, an open repair was required for relief of her hypertension and preservation of the renal function. An aortoaortic bypass (between the suprarenal and infrarenal abdominal aorta) with a Gore-Tex® ring reinforced tube (16 mm diameter, WL Gore & Associates Inc, USA), an orthotopic right renal autotransplantation and an aorto-left renal arterial bypass with autogenous saphenous vein grafts were performed. The operation was performed through an arch incision below the costal margin under general anesthesia. We obtained a satisfactory exposure through appropriate division and traction. The aorta coarctation extended approximately 7 cm. After heparinization, the descending aorta was clamped and the prosthetic graft was anastomosed to the aorta in an end-to-side fashion. The autogenous saphenous vein grafts were harvested. To revascularize the right renal artery, we performed an *ex vivo* right renal artery repair and orthotopic autotransplantation. The right renal artery and vein were detached and the kidney was cold perfused with University of Wisconsin (UW) solution at 4°C. The ureter was left intact. The proximal stenosis containing the stent was resected. The right kidney was then re-implanted into its original fossa. Then the right renal artery was reconstructed with the autogenous vein graft and re-implanted onto the prosthetic graft. And the right renal vein was re-implanted onto the inferior vena cava (Figure 2). The ischemic time for the right kidney was 1 hour and 45 minutes. The right kidney was fixed to the surrounding tissues to prevent migration or kinking. Next, the proximal stenosis of the left renal artery containing the stent was resected and the artery was re-implanted onto the prosthetic graft in an end-to-side fashion with autogenous saphenous vein graft.

The postoperative course was uneventful. Postoperative anticoagulant therapy consisted of low molecular weight heparin (4000 U/twice a day) for 2 weeks. The therapy was then switched to clopidogrel (75 mg/day) for 3 months and aspirin (100 mg/day) for 6 months.

**Figure 3:**
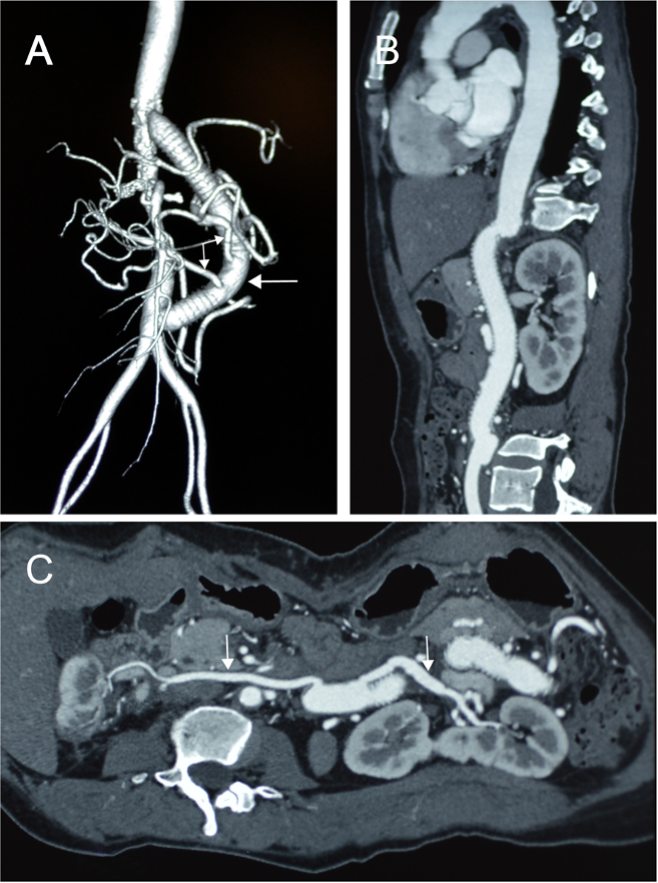
**Postoperative imaging showed that all the grafts were patent. A**, Postoperative three-dimensional computed tomography angiography showing the extra-anatomic bypass (right arrow) with the autogenous saphenous vein grafts (left arrows). **B**, Multi-planar reconstruction showing that the new prosthetic graft is patent. **C**, Curved-planar reformation of the computed tomography angiography showing that the saphenous vein grafts (two arrows) are patent.

Evaluation at one year showed that the patient was well, still on nifedipine on a daily basis, and had a blood pressure of 140/90 mmHg. 99mTc-DTPA renogram revealed that the GFR of the left and right kidney were 53.7 ml/min and 10.1 ml/min, respectively (total GFR 63.8 ml/min). The computed tomography angiography showed that all the grafts were patent and there were no abnormalities at the anastomosis (Figure 3).

## Discussion

We performed a one-stage revascularization for the patient. Percutaneous interventions are effective in relieving obstruction of the renal arteries. However, renal artery stenting has not generally been effective in treating Takayasu’s disease because of the severe vessel fibrosis [6]. In this patient, the symptoms of hypertension did not improve from renal artery stenting. In cases of complex disease affecting the renal or visceral arteries, bypass surgery is preferred [4]. Thus, severe stenoses of the visceral arteries and renal arteries in this patient justified an aggressive reconstructive approach. We previously reported a case of a 15-year-old boy with marked narrowing of the descending aorta, the orifice of the celiac artery and the superior mesenteric artery. The boy successfully underwent a bypass surgery [7].

To get a better exposure of the abdominal aorta and renal arteries, an arch incision was used below the costal margin. Nonetheless, reconstruction remained difficult because the right renal artery lied behind the inferior vena cava and the short right renal vein; moreover, the stenosis containing the stent needed resection, which rendered the reconstruction more difficult. Thus, right renal autotransplantation was indicated [8]. The preoperative renogram showed that the total GFR was 32.5 ml/min with the left kidney contributing 20.4 ml/min and the right kidney contributing 12.1 ml/min. To preserve the kidney function, right renal autotransplantation was preferred to a right radical nephrectomy. Multiple bypass surgery with reimplantation of the autogenous vein grafts onto the prosthetic graft was performed in bilateral renal artery revascularization. This anastomotic technique has been proved effective in our previous case report [7]. As the well-developed arc of Riolan provided enough flow to the superior mesenteric artery, the patient did not show any intestinal ischemic symptoms. Superior mesenteric artery revascularization did not appear to be necessary.

Postoperative hypertension in patients with MAS involves multiple systems [9]. In this patient, reduced arterial compliance and atrophied right kidney are expected to contribute to postoperative hypertension. The postoperative renogram revealed that left renal function was significantly improved and evaluation at one year showed that the patient’s blood pressure was satisfactory. Long-term follow-up of patients with operated MAS has shown that surgery does not offer a lasting cure and the rate of recurrence of hypertension is high and surgical re-intervention is often needed [10]. Long-term complications of bypass surgery include anastomotic aneurysm, congestive heart failure, graft deterioration, abdominal aortic aneurysms, renal failure, and other cardiovascular events [11]. Thus, lifelong follow-up is mandatory.

## Conclusion

We report a case of one-stage revascularization of severe MAS in a female patient who failed to respond to medical and endovascular therapy. In this case, multiple bypass surgery with reimplantation of autogenous vein grafts onto prosthetic graft is a feasible and effective procedure in renal artery revascularization. Besides, in cases of complex renal artery reconstruction, orthotopic autotransplantation may be the procedure of choice.

### Consent

Written informed consent was obtained from the patient for publication of this case report and any accompanying images. A copy of the written consent is available for review by the Editor of this journal.

## Abbreviations

MAS: Midaortic syndrome
GFR: Glomerular filtration rate
99mTc-DTPA: Technetium-99m-diethylenetriaminepentacetic acid
UW: University of Wisconsin.
